# Incretin Therapies for Patients with Type 2 Diabetes and Chronic Kidney Disease

**DOI:** 10.3390/jcm13010201

**Published:** 2023-12-29

**Authors:** Radica Z. Alicic, Joshua J. Neumiller

**Affiliations:** 1 Providence Medical Research Center, Providence Inland Northwest Health, 105 W. 8th Ave, Suite 250E, Spokane, WA 99204, USA; 2 Department of Medicine, University of Washington, Seattle, WA 98195, USA; 3 College of Pharmacy and Pharmaceutical Sciences, Washington State University, Spokane, WA 99164, USA

**Keywords:** diabetic kidney disease, GLP-1 receptor agonists, kidney outcomes, tirzepatide

## Abstract

Since the early 2000s, an influx of novel glucose-lowering agents has changed the therapeutic landscape for treatment of diabetes and diabetes-related complications. Glucagon-like peptide-1 (GLP-1) receptor agonists represent an important therapeutic class for the management of type 2 diabetes (T2D), demonstrating benefits beyond glycemic control, including lowering of blood pressure and body weight, and importantly, decreased risk of development of new or worsening chronic kidney disease (CKD) and reduced rates of atherosclerotic cardiovascular events. Plausible non-glycemic mechanisms that benefit the heart and kidneys with GLP-1 receptor agonists include anti-inflammatory and antioxidant effects. Further supporting their use in CKD, the glycemic benefits of GLP-1 receptor agonists are preserved in moderate-to-severe CKD. Considering current evidence, major guideline-forming organizations recommend the use of GLP-1 receptor agonists in cases of T2D and CKD, especially in those with obesity and/or in those with high cardiovascular risk or established heart disease. Evidence continues to build that supports benefits to the heart and kidneys of the dual GLP-1/glucose-dependent insulinotropic polypeptide (GIP) receptor agonist tirzepatide. Ongoing outcome and mechanistic studies will continue to inform our understanding of the role of GLP-1 and dual GLP-1/GIP receptor agonists in diverse patient populations with kidney disease.

## 1. Introduction

The upsurge in the number of cases of chronic kidney disease (CKD) in people with diabetes, a leading cause of kidney failure worldwide, is driven primarily by the global pandemics of obesity and diabetes [1,2,3]. When the development of persistent albuminuria and/or reduced glomerular filtration rate (GFR) is attributed to diabetes, the condition is referred to as chronic kidney disease (CKD) in diabetes, or diabetic kidney disease (DKD) [4]. DKD is associated with progression to kidney failure, requiring kidney replacement therapy, cardiovascular complications, increased risk of cardiovascular and all-cause mortality, and increased risk of infections and hospital admissions [5,6,7,8]. For close to two decades, the management of DKD was based on optimized control of blood glucose and blood pressure, and use of renin–angiotensin system (RAS) inhibitors in patients with hypertension and albuminuria [9,10,11,12,13]. This approach has since been transformed following unexpected findings from cardiovascular outcomes trials (CVOTs) mandated by the U.S. Food and Drug Administration (FDA) to ensure cardiovascular safety of new glucose-lowering agents entering the market [14]. Select CVOTs performed with agents from the glucagon-like peptide-1 (GLP-1) receptor agonist and sodium-glucose cotransporter-2 (SGLT-2) inhibitor classes reported reduced rates of cardiovascular events [15]. Secondary and exploratory findings from large GLP-1 receptor agonist CVOTs additionally suggested a beneficial effect of the class on worsening of kidney function [16,17,18,19,20]. Although originally approved to lower glucose in cases of type 2 diabetes (T2D), select GLP-1 receptor agonists have received expanded indications for cardiovascular risk reduction and treatment of obesity. Furthermore, GLP-1 receptor agonists have demonstrated many other favorable biological effects, including reductions in blood pressure and local and systemic markers of inflammation, with GLP-1 receptor agonists under active study for their effectiveness in other cardiovascular–kidney–metabolic (CKM) conditions [21,22]. Given the clear evidence of positive impact on glycemia, weight, and organ protection, GLP-1 receptor agonists are uniformly recommended by major professional society guidelines to improve outcomes for patients with diabetes and other CKM conditions [23,24,25,26,27,28,29]. Additionally, evidence continues to mount suggesting similar organ protective effects with the dual GLP-1/glucose-dependent insulinotropic polypeptide (GIP) agonist, tirzepatide [30,31]. While a dedicated trial examining the effects of tirzepatide on kidney outcomes is ongoing, a post hoc analysis of completed clinical trials indicated that reductions in albuminuria and estimated glomerular filtration rate (eGFR) decline with tirzepatide versus comparators [32], thus suggesting potential kidney benefits with the first agent in this novel class of incretin therapies. This review provides a succinct discussion of the biology of incretins, proposed mechanisms of action of GLP-1 receptor agonists, clinical evidence to date demonstrating kidney benefits with GLP-1 and dual GLP-1/GIP receptor agonists, and overview of current recommendations for GLP-1 receptor agonist use in patients with T2D and DKD.

## 2. Incretin Biology: An Overview

**Figure 1:**
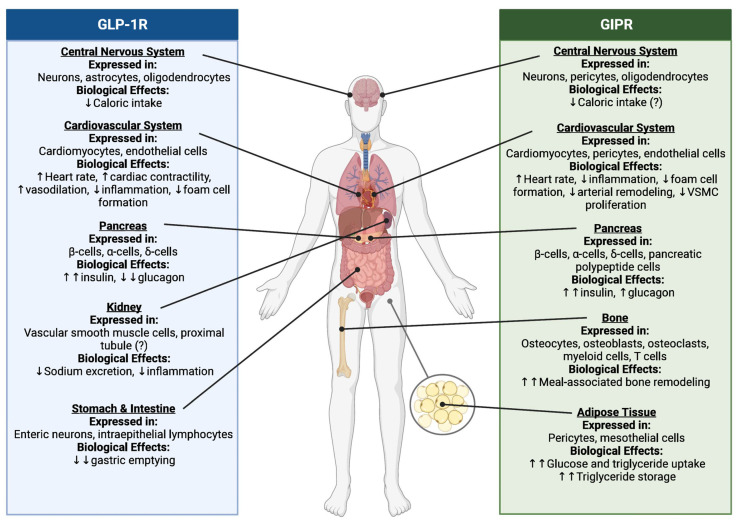
Tissue distribution of glucagon-like peptide-1 (GLP-1) and glucose-dependent insulinotropic polypeptide (GIP) receptors and proposed biological actions. Abbreviations: VSCM, vascular smooth muscle cells. From: Reprinted under the Creative Commons Attribution-NonCommercial-NoDerivatives License 4.0 (CCBY-NC-ND) from [41].

The **IN**testine se**CRET**ion of **IN**sulin (**INCRETIN**) effect, describing insulin secretion in the postprandial state in response to oral intake of glucose, was first described in the first half of the 20th century. Specifically, the incretin effect is driven by insulin secretory effects of the incretin hormones GLP-1 and GIP [33,34]. GLP-1 and GIP are secreted by enteroendocrine L cells in the terminal ileum and colon, and K cells in the duodenum and jejunum, respectively [35,36]. In addition to oral nutrient intake, GLP-1 release is stimulated by microbiomic products, immune-cell-derived cytokines, and neuroendocrine modulators, indicating its central role in the interface between metabolic processes and immune response [37,38]. Postprandial effects of both peptides are short lived (~4–7 min) because they are rapidly inactivated by dipeptidyl peptidase-4 (DPP-4) [39]. Endogenous GLP-1 and GIP act via specific receptors broadly expressed in multiple organs (Figure 1) [40,41]. Both GLP-1 and GIP demonstrate insulinotropic effects, with opposing effects on pancreatic secretion of glucagon, which is suppressed with GLP-1 and promoted by GIP [40,42]. Another important biological role of endogenous incretin hormones is weight regulation. GLP-1 facilitates weight loss by slowing motility of the upper gastrointestinal tract, decreasing gastrointestinal secretions, and modulating satiety through actions in the central nervous system [43,44]. GIP enhances blood flow in adipose tissue, increases insulin sensitization, and promotes energy expenditure through mechanisms in the central nervous system [45]. Only GLP-1 receptors have been identified in the kidneys (Figure 1) [41]. The exact location of GLP-1 receptors in the kidneys is still under investigation. To date, *GLP-1 receptor* mRNA has been detected in different kidney vascular structures, proximal tubular cells, and resident and infiltrating inflammatory cells [46,47,48,49]. Accordingly, important organ protective mechanisms of GLP-1 receptor agonist therapy are related to their anti-inflammatory and antioxidative effects [38,50,51,52,53,54]. Evidence shows that GLP-1 receptor activation downregulates multiple pro-inflammatory pathways including protein kinase A/activator of transcription 3 (PKA/STAT3), phosphoinositide 3-kinases/protein kinase B (PI3K/AKT), and mitogen-activated protein kinase/nuclear factor-κB (MAPK/NF-κB) [54,55,56]. In a mouse model, liraglutide ameliorated oxidative stress through increasing cyclic adenosine monophosphate (cAMP) levels and PKA activity, and reducing nicotinamide adenine dinucleotide phosphate oxidase (NADPH oxidase) without changes in insulin secretion or glucose tolerance [47]. Furthermore, in healthy volunteers and experimental models, GLP-1 receptor activation promotes natriuresis and diuresis via inhibition of sodium-hydrogen exchanger 3 (NHE3)-mediated Na+/H+ exchange in the proximal tubule. This action partially explains the blood pressure-lowering effect of GLP-1 receptor agonists [48,49].

## 3. Proposed Mechanisms of Benefit in Diabetic Kidney Disease

Structural and functional changes are consistent with DKD results from ongoing exposure to multilayered, self-perpetuating metabolic (insulin resistance, hyperinsulinemia, hyperglycemia, obesity), hemodynamic (inappropriate RAS activation, increased glucose and sodium chloride uptake, hyperfiltration, glomerular hypertension), and pro-inflammatory/pro-fibrotic (activation of major immune pathways, oxidative stress, cytokine production, and fibrotic factors) processes [57,58,59,60,61].

GLP-1 receptor agonists positively modify multiple risk factors, including glycemia, body weight, and systolic blood pressure, in addition to reducing LDL cholesterol and triglyceride levels [62,63,64]. In most studies, GLP-1 receptor agonism reduced glycated hemoglobin A1C (HbA1c) by 0.8–1.5% (8–15 mmol/mol), systolic blood pressure by 3–4 mmHg, and promoted an approximate mean 3 kg weight loss [65]. Indeed, both liraglutide and injectable semaglutide have received approval in the United States for the treatment of obesity irrespective of the presence of diabetes [66]. The glucose-lowering benefits of GLP-1 receptor agonists are preserved in advanced CKD and have demonstrated efficacy and safety in large clinical trials with an eGFR of 15 mL/min/1.73 m^2^ [67]. It is important to note that data from several studies and meta-analyses indicate organ benefits from mechanisms other than direct metabolic effects. For instance, in the Assessment of Weekly AdministRation of LY2189265 (Dulaglutide) in Diabetes 7 (AWARD-7) trial, beneficial effects on eGFR decline were observed with dulaglutide treatment when compared to insulin glargine, despite similar blood glucose and blood pressure control between treatment groups [67]. Furthermore, an exploratory mediation analysis of liraglutide and semaglutide trials estimated that improvements in glycemia, blood pressure, and body weight mediated observed effects on doubling of serum creatinine, reaching an eGFR < 45 mL/min/1.73 m^2^, or progression to kidney failure by only 10–25% [21].

**Figure 2:**
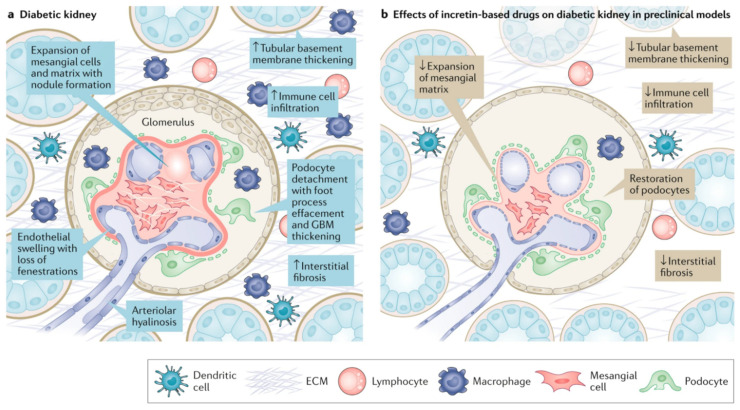
Incretin effects on structural kidney damage observed in diabetic kidney disease. (**a**) Histological manifestations of diabetic kidney disease include glomerular hypertrophy with expansion of the mesangium by matrix and mesangial cells; mesangial matrix accumulation with the formation of nodules (Kimmelstiel–Wilson nodules) and focal to global glomerulosclerosis; thickening of the glomerular basement membrane (GBM); podocyte foot process fusion, effacement, and loss; tubular basement membrane thickening with interstitial inflammation, fibrosis, and immune cell infiltration (including macrophages, lymphocytes, and polymorphonuclear leukocytes); and arteriolar hyalinosis. (**b**) Treatment with incretin-based therapies can ameliorate the structural changes in the kidneys that are induced by diabetes, at least in part, through anti-inflammatory and antifibrotic effects. Abbreviations: ECM, extracellular matrix. From: Reprinted with permission from [70].

In individuals with diabetes and obesity, GLP-1 receptor agonist therapy reduced levels of inflammatory macrophages as well as multiple inflammatory and oxidative markers (e.g., interleukin-6, interleukin-1β, monocyte chemoattractant protein-1, adhesion molecules, prostaglandins, serum amyloid A, tumor necrosis factor-α, toll-like receptors) [41,68]. Antioxidative and anti-inflammatory effects were demonstrated in multiple rodent models of DKD, where GLP-1 receptor agonist exposure was associated with suppression of oxidative stress, inflammatory cell infiltration of kidney, and reduced activation of proinflammatory cytokines and profibrotic factors [54,55,56,69]. Moreover, in rodent and cell culture models, liraglutide, exendin-4, and GLP-1 reduced pathological findings characteristic of DKD, including mesangial expansion and cell proliferation, glomerular hypertrophy, and tubulointerstitial and glomerular fibrosis [54,55,56,70]. These changes correlate with amelioration of structural (e.g., reduction in kidney hypertrophy, mesangial matrix expansion, loss of podocytes and glomerular basement membrane thickness) and functional changes (e.g., reduction in albuminuria) of DKD (Figure 2) [54,55,56,69,70]. Other proposed beneficial effects of GLP-1 receptor agonists in other tissues include pulmonary protective effects and beneficial effects on gut microbiome composition [71]. In summary, mounting evidence points to beneficial GLP-1 receptor agonist effects on inflammation, oxidative stress, and fibrosis as crucial mechanisms behind the kidney protective actions of this class [72,73,74]. Similarly, within the cardiovascular system, GLP-1 receptor agonism also reduces inflammation, increases endothelial proliferation and angiogenesis, and promotes vasodilation, plaque stability, and improved cardiac perfusion [50,75,76,77].

## 4. GLP-1 Receptor Agonist Cardiovascular Outcome Trials

The primary composite endpoint of most major CVOTs with agents from the GLP-1 receptor agonist class is a three-point major adverse cardiovascular event (MACE) outcome, consisting of time to first event of either cardiovascular death, non-fatal myocardial infarction, or non-fatal stroke [15]. CVOTs completed with liraglutide, injectable semaglutide, and dulaglutide have reported benefits over placebos for their primary MACE outcome, leading to expanded cardiovascular indications for these agents by the U.S. FDA [16,17,19]. While primary kidney outcome trials are still ongoing, secondary and exploratory kidney outcomes derived from CVOT data have provided hypothesis-generating data suggesting improved kidney outcomes with agents from the GLP-1 receptor agonist class, driven primarily by preventing development of new, or reducing existing, albuminuria [16,17,19,78]. A post hoc analysis of prespecified secondary kidney outcomes in the SUSTAIN-6 and LEADER trials showed a reduced risk of progression of albuminuria, increased likelihood of regression in albuminuria, and slower annual rate of eGFR decline in patients treated with semaglutide and liraglutide when compared with a placebo [79,80]. The effect on eGFR decline was more pronounced in the subpopulation with eGFR < 60 mL/min/1.73 m^2^ (mean annual estimated treatment difference (ETD) between slopes of 1.62 mL/min/1.73 m^2^ and 0.67 mL/min/1.73 m^2^ favoring semaglutide and liraglutide groups relative to placebo, respectively) [80]. A 2019 meta-analysis that included over 56,000 participants enrolled across seven GLP-1 receptor agonist CVOTs reported that GLP-1 receptor agonist treatment reduced the risk of MACE by 12% (hazard ratio (HR): 0.88; 95% confidence interval (CI): 0.82–0.94; *p* < 0.001), all-cause mortality by 12% (HR: 0.88, 95% CI: 0.83–0.95; *p* = 0.001) and kidney outcomes (development of new-onset macroalbuminuria, decline in eGFR (or increase in creatinine), progression to end-stage kidney disease, or death attributable to kidney causes) by 17% (HR: 0.83, 95% CI: 0.78–0.89; *p* < 0.001) [81]. A subsequent 2021 meta-analysis included new data from the efpeglenatide CVOT, the AMPLITUDE-O trial [82]. The meta-analysis included over 60,000 patients enrolled in CVOTs completed with lixisenatide, liraglutide, injectable semaglutide, exenatide, albiglutide, dulaglutide, oral semaglutide, and efpeglenatide (currently in phase 3 clinical trials). Overall, treatment with GLP-1 receptor agonists in patients with T2D reduced the risk of three-point MACE by 14% (HR: 0.88; 95% CI: 0.82–0.94; *p* = 0.0001), and the composite kidney outcome consisting of development of macroalbuminuria, worsening of kidney function (based on eGFR change), kidney replacement therapy, or death due to kidney disease by 21% (HR: 0.79; 95% CI: 0.73–0.87) with treatment effect at least as large in patients with eGFR < 60 mL/min/1.73 m^2^ [83]. This finding is clinically important when considering the enhanced cardiovascular risk in persons with CKD.

An additional benefit of GLP-1 receptor agonist therapy in cases of T2D and CKD comes from the AWARD-7 trial with dulaglutide [67]. AWARD-7 was a glycemic efficacy and safety trial that enrolled participants with moderate-to-severe CKD (mean eGFR 38 mL/min/1.73 m^2^, inclusive of participants with baseline eGFR down to 15 mL/min/1.73 m^2^). At trial end, participants randomized to dulaglutide experienced less eGFR decline from baseline and attenuation of albuminuria when compared to participants randomized to treatment with insulin glargine. Importantly, reduction in the rate of eGFR decline was maintained in those with urinary albumin-to-creatinine ratio (UACR) > 300 mg/g (macroalbuminuria) at high-risk of kidney disease progression [67,84]. A summary of exploratory kidney outcomes and ongoing mechanistic and kidney outcome trials is provided in Table 1 [16,17,18,19,67,78,82,85,86,87,88].

## 5. Dual GLP-1/Glucose-Dependent Insulinotropic Polypeptide (GIP) Receptor Agonist: Evidence to Date

Dual GLP-1/GIP receptor agonism with tirzepatide demonstrates impressive reduction in principal CKD risk factors including blood pressure, blood glucose, and weight when compared to GLP-1 receptor agonism alone [89,90]. A meta-analysis of pooled data from seven tirzepatide trials showed that tirzepatide treatment resulted in a median dose-related blood pressure reduction of −4.20 mmHg (95% CI: −5.17 to −3.23) for a 5 mg dose, −5.34 mmHg (95% CI: −6.31 to −4.37) for a 10 mg dose, and −5.77 mmHg (95% CI: −6.73 to −4.81) for a 15 mg dose [91]. Furthermore, when compared with placebos and other glucose-lowering agents, tirzepatide demonstrated dose-dependent superiority in lowering HbA1C (−1.62% to −2.06% vs. placebo, −0.29% to −0.92% vs. GLP-1 receptor agonists, and −0.70% to −1.09% vs. basal insulin), and reducing body weight (reductions vs. GLP-1 receptor agonists ranged from 1.68 kg with 5 mg tirzepatide to 7.16 kg with 15 mg tirzepatide) [92]. More recent weight loss trials in participants with obesity have demonstrated robust weight loss with tirzepatide therapy in patients with and without diabetes [93,94], with 79–83% of participants with obesity and T2D treated with tirzepatide in the SURMOUNT-2 trial achieving ≥5% weight loss from baseline, compared to 32% receiving a placebo [94]. The first report on direct effects of dual GLP-1/GIP receptor agonism on the kidneys comes from a prespecified exploratory analysis of the Tirzepatide Versus Insulin Glargine in Type 2 Diabetes and Increased Cardiovascular Risk (SURPASS-4) study [32]. The analysis reported that when compared with insulin glargine, tirzepatide use was associated with a slower rate of eGFR decline, lower albuminuria, and significantly reduced occurrence of the composite kidney endpoint of eGFR decline ≥40% from baseline, end-stage kidney disease, death due to kidney failure, or new-onset macroalbuminuria (HR: 0.58; 95% CI: 0.43–0.80) [32]. The largest observed beneficial effect was on reducing albuminuria. This report is of particular interest given that 25% of participants were also receiving an SGLT2 inhibitor [32], suggesting an additional kidney benefit of these guideline-directed medical therapies when used in combination. The Tirzepatide Study of Renal Function in People With Overweight or Obesity and Chronic Kidney Disease With or Without Type 2 Diabetes: Focus on Kidney Hypoxia in Relation to Fatty Kidney Disease Using Multiparametric Magnetic Resonance Imaging (TREASURE-CKD; NCT05536804) trial is currently enrolling participants and will examine the effect of tirzepatide on primary kidney outcomes and explore potential mechanisms of kidney benefits with tirzepatide.

## 6. Current Guidance on GLP-1RA Use in T2D and DKD

Multiple GLP-1 receptor agonists and one dual GLP-1/GIP receptor agonist are available for clinical use (Table 2) [95,96,97,98,99,100,101,102]. In consideration of evidence showing robust beneficial effects of these agents on glycemia, weight, and outcomes, guidelines for the management of T2D have evolved dramatically in recent years. The American Diabetes Association (ADA), the American Association of Clinical Endocrinologists (AACE), the European Association for the Study of Diabetes (EASD), the European Society of Cardiology (ESC), and Kidney Disease: Improving Global Outcomes (KDIGO) all now recommend GLP-1 receptor agonists for glycemic control and cardiovascular risk reduction in patients with T2D with or without DKD [23,24,25,26,27,28,29]. GLP-1 receptor agonists are universally recommended as an option in T2D to reduce HbA1c and to assist with achieving and maintaining individualized weight management goals [23,25,26,27,29]. ADA additionally recommends a GLP-1 receptor agonist in preference to insulin for patients with T2D who require greater glucose lowering than can be achieved with oral glucose-lowering agents [25]. In patients with T2D and DKD, ADA and KDIGO recommend the addition of a GLP-1 receptor agonist in those unable to achieve individualized glycemic goals despite first-line treatment with an SGLT2 inhibitor and metformin [23]. This recommendation is based in part on emerging evidence for kidney benefits observed in large CVOTs and the preserved glucose-lowering effects of GLP-1 receptor agonists in advanced CKD.

Guidelines also emphasize the importance of counseling patients about common side effects as well as risk mitigation strategies to ensure safe medication use. The most common dose-limiting side effects with GLP-1 and dual GLP-1/GIP receptor agonists are related to their biological function and are gastrointestinal (GI) tract related (e.g., nausea, decreased appetite, vomiting, and diarrhea) [23]. Generally, GI side effects improve after several weeks of therapy at a given dose, and can be minimized by initiating with a lower dose and slowly titrating the dose upward [103]. While GLP-1 receptor agonists and tirzepatide do not cause hypoglycemia when used as monotherapy, there is a risk of additive hypoglycemia when combined with sulfonylureas or insulin, with close monitoring and downward dose adjustment or withdrawal of background hypoglycemic agents (e.g., sulfonylureas, insulin) recommended to avoid unnecessary hypoglycemic events [23].

## 7. Future Directions

The kidney effects of GLP-1 receptor agonists and tirzepatide and their mechanisms of action are areas of active investigation. The Effect of Semaglutide Versus Placebo on the Progression of Renal Impairment in Subjects With Type 2 Diabetes and Chronic Kidney Disease (FLOW study; NCT03819153) is the first dedicated kidney outcome trial with a GLP-1 receptor agonist that was halted prematurely due to meeting prespecified criteria for efficacy, with detailed results anxiously expected in 2024. The ongoing Semaglutide Cardiovascular Outcomes Trial in Patients With Type 2 Diabetes (SOUL trial; NCT03914326) trial is examining the efficacy of oral semaglutide for a combined cardiovascular and kidney outcome in participants with T2D and established cardiovascular disease or CKD. A complement to the FLOW and SOUL trials, the Renal Mode of Action of Semaglutide in Patients With Type 2 Diabetes and Chronic Kidney Disease (REMODEL trial; NCT04865770), is exploring possible mechanisms of kidney benefits with semaglutide. The Tirzepatide Study of Renal Function in People With Overweight or Obesity and Chronic Kidney Disease With or Without Type 2 Diabetes: Focus on Kidney Hypoxia in Relation to Fatty Kidney Disease Using Multiparametric Magnetic Resonance Imaging (TREASURE-CKD; NCT05536804) is currently enrolling participants and will examine the effect of tirzepatide on primary kidney outcomes and potential mechanisms of action. To help address the question of potential GLP-1 receptor agonist benefits in people with type 1 diabetes, the REMODEL-1 study will evaluate CKD outcomes in people with type 1 diabetes. The results of these trials will provide further clarity on the benefits and clinical role of GLP-1 and dual GLP-1/GIP receptor agonists in diverse populations with DKD.

## 8. Conclusions

GLP-1 receptor agonists and the dual GLP-1/GIP receptor agonist tirzepatide have emerged as potent glucose-lowering and weight loss agents with additional benefits of cardiovascular and kidney risk reduction. Evidence suggests that the organ protective effects of these agents extend beyond their metabolic benefits, with GLP-1 receptor agonism associated with anti-inflammatory and antifibrotic effects in the heart and kidneys. While current guidelines widely recommend GLP-1 receptor agonists for the treatment of CKM conditions, ongoing research will continue to inform the role of GLP-1 and dual GLP-1/GIP receptor agonists in diverse populations with kidney disease.

## Figures and Tables

**Table 1:** Kidney outcomes from completed and ongoing GLP-1 receptor agonist and dual GLP-1/GIP agonist clinical trials.

Trial	Treatment Arms	Duration of Follow-Up/Trial Status	Study Population Characteristics	Primary, Secondary, and/or Exploratory Kidney Outcomes
**Completed Trials**				
LEADER [16,85]	Liraglutide 0.6 to 1.8 mg daily vs. placebo	Median 3.8 years	T2D Mean HbA1c > 7% Established CVD in 81% of participants Mean BP: 167/77 mmHg Mean BMI: 32.5 ± 6.3 kg/m^2^ Kidney function: o 21% with eGFR 30–59 o 2% with eGFR < 30	**Secondary microvascular outcome:** lower rate of DKD events in the liraglutide treatment group (1.5 events per 100 patient-years) compared with placebo (1.9 events per 100 patient-years) (HR 0.84, 95% CI 0.73–0.97; *p* = 0.02) **Prespecified secondary kidney outcome:** reduction in the composite kidney outcome of new-onset persistent macroalbuminuria, persistent doubling of the serum creatinine level, kidney failure or death due to kidney disease in the liraglutide treatment group (HR 0.78; 95% CI 0.67–0.92; *p* = 0.003)
EXCEL [18,86]	Exenatide 2 mg once weekly vs. placebo	Median 3.2 years	T2D Mean HbA1c 8.1% Established CVD in 73% of participants Mean systolic BP: 135 mmHg Mean BMI: 32.7 kg/m^2^ Kidney function: o Mean eGFR 76 o 18% with eGFR < 60	**Prespecified secondary kidney outcomes:** o New macroalbuminuria occurred in 2.2% and 2.5% of participants in the exenatide and placebo groups, respectively (HR 0.87; 95% CI 0.70–1.07) o No difference between treatment groups for kidney composite 1 (40% eGFR decline, kidney replacement, or kidney disease-related death) (HR 0.88; 95% CI 0.74–1.05; *p* = 0.16) o No difference between treatment groups for kidney composite 2 (40% eGFR decline, kidney replacement, kidney disease-related death, or macroalbuminuria) (HR 0.88; 95% CI 0.76–1.01; *p* = 0.07)
ELIXA [78,87]	Lixisenatide 10 to 20 mcg daily vs. placebo	Median 2.1 years	T2D with recent acute coronary syndrome Mean HbA1c 7.7% Mean systolic BP: 130 mmHg Mean BMI: 30.2 kg/m^2^ Kidney function: o Mean eGFR 77 o Median UACR 10.4 mg/g	**Exploratory analyses:** o Placebo-adjusted LSM percentage change in UACR from baseline was −39.2% (95% CI −68.5 to −9.8; *p* = 0.04) in participants with macroalbuminuria o Reduced risk of new-onset macroalbuminuria compared with placebo when adjusted for baseline HbA1c (HR 0.81; 95% CI 0.66–0.99; *p* = 0.04) or when adjusted for baseline and on-trial HbA1c (HR 0.82; 95% CI 0.67–0.99; *p* = 0.049)
SUSTAIN-6 [17]	Semaglutide 0.5 to 1 mg weekly vs. placebo	Median 2.1 years	T2D HbA1c > 7% Mean body weight: 92.1 kg Prior CVD in 83% of participants Mean BP: 136/77 mmHg Kidney function: o 25% eGFR 30–59 o 3% eGFR ≤ 30	**Prespecified secondary outcome:** o New or worsening nephropathy (persistent UACR > 300 mg/g, doubling of serum creatinine or eGFR < 45 mL/min/1.73 m^2^, need for kidney replacement therapy) occurred in 3.8% and 6.1% of participants in the semaglutide and placebo groups, respectively (HR 0.64; 95% CI 0.46–0.88; *p* = 0.005)
AWARD-7 [67,88]	Dulaglutide 0.75 to 1.5 mg weekly vs. insulin glargine daily	52 weeks (treatment trial)	T2D HbA1c 7.5–10.5% Mean BP: 137/75 mmHg Mean BMI: 32.5 kg/m Kidney function: o Mean eGFR 38 o 26% eGFR 45–60 o 35% eGFR 30–45 o 31% eGFR < 30 o 29% UACR > 30 mg/g o 46% UACR > 300 mg/g	**Prespecified secondary outcomes:** o eGFR decline (mL/min) was −3.3 with insulin glargine, −0.7 with dulaglutide 0.75 mg, and −0.7 with dulaglutide 1.5 mg o eGFR decline (mL/min) in UACR >300 mg/g group was −5.5 in the insulin glargine group, −0.7 in the dulaglutide 0.75 mg group, and −0.5 in the dulaglutide 1.5 mg group o UACR reduction: ▪ −13% in the insulin glargine group, −12.3% in the dulaglutide 0.75 mg group, and −29% in the dulaglutide 1.5 mg group o Composite endpoint of kidney failure or >40% eGFR decline: ▪ 5.2% in dulaglutide 1.5 mg group ▪ 10.8% in insulin glargine group (*p* = 0.038)
REWIND [19]	Dulaglutide 1.5 mg weekly vs. placebo	Median 5.4 years	T2D Mean HbA1c 7.3% in dulaglutide group; 7.4% in placebo group Prior CVD in 31% of participants Mean BP 137/78 mmHg Mean BMI 32 kg/m^2^ Kidney function: o Mean eGFR 75 o Mean UACR 16 mg/g	**Secondary composite kidney outcome:** o Defined as development of a urinary albumin-to-creatinine ratio > 300 mg/g in those with a lower baseline concentration, a sustained 30% or greater decline in eGFR or chronic kidney replacement therapy occurred in 20% of participants in the placebo group vs. 17% in the dulaglutide group (HR 0.85; 95% CI 0.77–0.93; *p* = 0.0004)
AMPLITUDE-O [82]	Efpeglenatide 4 to 6 mg weekly vs. placebo	Median 1.8 years	T2D Mean HbA1c 8.9% Mean BP 135/77 Prior CVD in 89.6% of participants Mean BMI 32 kg/m^2^ Kidney function: o 31.6% eGFR < 60 o Median UACR 28.3 mg/g o 21.8% with CVD and eGFR < 60	**Secondary composite kidney outcome:** o Defined as development of macroalbuminuria, increase in albumin-to-creatinine ratio of >30%, a sustained ≥40% decline in eGFR, end-stage kidney disease, or death due to any cause occurred in 18.4% of participants in the placebo group and 13% of participants in the efpeglenatide group (HR 0.68; 95% CI 0.57–0.79; *p* < 0.001)
**Ongoing Trials**				
FLOW (NCT03819153)	Weekly semaglutide vs. standard of care	Results pending	T2D HbA1c 6.5% to 12% High CV risk UACR 300 to 5000 mg/g eGFR 30 to ≤90 mL/min/1.73 m^2^	**Primary kidney composite outcome:** o Defined as persistent eGFR decline of ≥50% from trial start, reaching kidney failure, death from kidney disease, or death from CVD
SOUL (NCT03914326)	Oral semaglutide 3 mg, 7 mg, or 14 mg daily	In progress	T2D HbA1c 6.5% to 10.0% Established CVD	**Secondary composite outcome:** o Defined as CV or kidney death, onset of persistent ≥ 50% eGFR decline, onset of persistent eGFR < 15 mL/min/1.73 m^2^, or initiation of kidney replacement therapy
TREASURE-CKD (NCT05536804)	Tirzepatide once weekly vs. standard of care	In progress	With and without T2D BMI ≥ 27 kg/m^2^ eGFR ≥ 30 to ≤60 mL/min/1.73 m^2^ or eGFR ≥ 30 to ≤75 mL/min/1.73 m^2^ if UACR > 30 mg/g	**Primary outcome:** o Change from baseline in kidney oxygenation in participants with or without T2D
REMODEL (NCT04865770)	Semaglutide once weekly vs. standard of care	In progress	T2D HbA1C < 9% eGFR ≥ 30 to ≤75 mL/min/1.73 m^2^ UACR > 20 to <5000 mg/g	**Primary outcome:** o Change in kidney oxygenation and inflammation assessed by MRI

**Abbreviations**: BMI, body mass index; BP, blood pressure; CI, confidence interval; CV, cardiovascular; CVD, cardiovascular disease; DKD, diabetic kidney disease; eGFR, estimated glomerular filtration rate; HbA1C; glycated hemoglobin A1c; HR, hazard ratio; LSM, least-squares mean; MRI, magnetic resonance imaging; T2D; type 2 diabetes mellitus; UACR, urinary albumin-to-creatinine ratio.

**Table 2:** Key administration and dosing information for currently available GLP-1 and dual GLP-1/GIP receptor agonists [95,96,97,98,99,100,101,102].

Agent	Administration Frequency	Indication(s)	Recommended Kidney Dose Adjustment
**GLP-1 Receptor Agonists**			
Exenatide	Twice daily	Adjunct to diet and exercise to improve glycemic control in adults with T2D	Not recommended with CrCl < 30 mL/min Caution recommended with initiating or escalating the dose with CrCl 30–50 mL/min
Liraglutide	Once daily	Adjunct to diet and exercise to improve glycemic control in patients ≥ 10 years with T2D To reduce the risk of MACE in adults with T2D and established CVD	No dosage adjustments recommended
Lixisenatide	Once daily	Adjunct to diet and exercise to improve glycemic control in adults with T2D	Not recommended with eGFR < 15 mL/min/1.73 m^2^
Dulaglutide	Once weekly	Adjunct to diet and exercise to improve glycemic control in adults and pediatric patients ≥ 10 years old with T2D To reduce the risk of MACE in adults with T2D and established CVD or multiple CV risk factors	No dosage adjustments recommended
Exenatide XR	Once weekly	Adjunct to diet and exercise to improve glycemic control in adults and pediatric patients ≥ 10 years old with T2D	Not recommended with eGFR < 45 mL/min/1.73 m^2^ or ESKD
Semaglutide	Once weekly (SubQ)	Adjunct to diet and exercise to improve glycemic control in adults with T2D To reduce the risk of MACE in adults with T2D and established CVD	No dosage adjustments recommended
	Once daily (Oral)	Adjunct to diet and exercise to improve glycemic control in adults with T2D	
**Dual GLP-1/GIP Receptor Agonist**			
Tirzepatide	Once weekly	Adjunct to diet and exercise to improve glycemic control in adults with T2D	No dosage adjustments recommended

**Abbreviations:** CrCl, creatinine clearance; CV, cardiovascular; CVD, cardiovascular disease; eGFR, estimated glomerular filtration rate; GIP, glucose-dependent insulinotropic peptide; GLP-1, glucagon-like peptide-1; MACE, major adverse cardiovascular events; SubQ, subcutaneous; T2D, type 2 diabetes; XR, extended release.
